# Screening of Mono-, Di- and Trivalent Cationic Dopants for the Enhancement of Thermal Behavior, Kinetics, Structural, Morphological, Surface and Magnetic Properties of CoFe_2_O_4_-SiO_2_ Nanocomposites

**DOI:** 10.3390/ijms24119703

**Published:** 2023-06-02

**Authors:** Thomas Dippong, Erika Andrea Levei, Ioan Petean, Iosif Grigore Deac, Raluca Anca Mereu, Oana Cadar

**Affiliations:** 1 Faculty of Science, Technical University of Cluj-Napoca, 76 Victoriei Street, 430122 Baia Mare, Romania; 2 INCDO-INOE 2000, Research Institute for Analytical Instrumentation, 67 Donath Street, 400293 Cluj-Napoca, Romania; 3 Faculty of Chemistry and Chemical Engineering, Babes-Bolyai University, 11 Arany Janos Street, 400084 Cluj-Napoca, Romaniaraluca.mereu@ubbcluj.ro (R.A.M.); 4 Faculty of Physics, Babes-Bolyai University, 1 Kogalniceanu Street, 400084 Cluj-Napoca, Romania

**Keywords:** cobalt ferrite, silica matrix, doping, calcination, magnetic behavior

## Abstract

CoFe_2_O_4_ is a promising functional material for various applications. The impact of doping with different cations (Ag^+^, Na^+^, Ca^2+^, Cd^2+^, and La^3+^) on the structural, thermal, kinetics, morphological, surface, and magnetic properties of CoFe_2_O_4_ nanoparticles synthesized via the sol-gel method and calcined at 400, 700 and 1000 °C is investigated. The thermal behavior of reactants during the synthesis process reveals the formation of metallic succinates up to 200 °C and their decomposition into metal oxides that further react and form the ferrites. The rate constant of succinates’ decomposition into ferrites calculated using the isotherms at 150, 200, 250, and 300 °C decrease with increasing temperature and depend on the doping cation. By calcination at low temperatures, single-phase ferrites with low crystallinity were observed, while at 1000 °C, the well-crystallized ferrites were accompanied by crystalline phases of the silica matrix (cristobalite and quartz). The atomic force microscopy images reveal spherical ferrite particles covered by an amorphous phase, the particle size, powder surface area, and coating thickness contingent on the doping ion and calcination temperature. The structural parameters estimated via X-ray diffraction (crystallite size, relative crystallinity, lattice parameter, unit cell volume, hopping length, density) and the magnetic parameters (saturation magnetization, remanent magnetization, magnetic moment per formula unit, coercivity, and anisotropy constant) depend on the doping ion and calcination temperature.

## 1. Introduction

Due to their potential use in various technological fields such as gas sensors, catalysis, magnetic imaging, high-density recording media, ferrofluids, microwave devices, magnetic data storage, and transformer cores, spinel ferrites have attracted considerable attention [1,2,3]. Depending on the cation distribution on A and B sites, spinel ferrites display ferrimagnetic, antiferromagnetic, spin-glass, or paramagnetic behavior [1]. Among them, cobalt ferrite (CoFe_2_O_4_) presents superparamagnetic behavior, high chemical stability, good mechanical hardness, moderate saturation magnetization (*M_S_*), high coercivity (*H_C_*), positive anisotropy constant (*K*), significant electrical resistance, low eddy current losses and low production costs [4,5,6,7]. CoFe_2_O_4_ is a promising candidate for magnetic resonance imaging, drug delivery, tomography, tissue imaging ferrofluids, recording heads, magnetic sensors, microwave devices, magnetic refrigeration, catalysis, pigments, gas detectors, environmental remediation, etc. [4,8,9,10,11].

By doping a small amount of an external ion in the composition of CoFe_2_O_4_, the physicochemical properties can be tailored without undesired phase transformation [3]. The properties of CoFe_2_O_4_ can be easily tailored as they depend on the synthesis method, composition, doping ions, and particle size distribution [4,10,11]. The properties of CoFe_2_O_4_ doped with divalent (Mg^2+^, Cu^2+^, Ni^2+^, Zn^2+^, Mn^2+^) and trivalent (La^3+^, Ru^3+^, Gd^3+^, Al^3+^) ions were extensively studied [12,13,14,15,16,17,18], while the doping with monovalent ions received less attention. The most common monovalent ion for ferrite doping is Ag^+^ due to its antibacterial activity against different pathogens [19]. Doping CoFe_2_O_4_ with non-magnetic Na^+^ ions resulted in good chemical stability but decreased magnetic properties [20]. Non-magnetic metal ions such as Cd^2+^ are particularly important because they do not display a magnetic moment but may change the magnetic properties by disturbing the magnetic moment’s equilibrium in the ferrite composition [3]. The photocatalytic and magneto-optical properties of transition metal-doped CoFe_2_O_4_ have received extensive research for future use in electronics, telecommunication, environmental and biomedical applications, catalysis, etc. [21,22,23,24]. The properties of CoFe_2_O_4_, especially those related to magnetic behavior, conductivity, and catalytic activity, are enhanced by doping with noble metal ions [25]. Additionally, Ag nanoparticles play a non-magnetic spacer role that allows the tuning of the dipolar magnetic interactions between the magnetic nanoparticles [5]. The Ag doping in CoFe_2_O_4_ provides good physical properties and antimicrobial activity, but its preparation requires complex (i.e., surface linkers, reducing and protecting agents) and time-consuming procedures that make it inappropriate for large-scale production [19,26,27]. So, a simple route for its large-scale preparation is still being developed. There is an ongoing debate on spinel ferrites doping with La^3+^, as some studies reported that the effect on coercivity and superexchange occurs most probably due to the location of La^3+^ ions on the sample’s surface or interstitial sites. However, the most often proposed mechanism is the partial replacement of Fe^3+^ by La^3+^ in B sites, resulting in unit cell expansion, increased structural distortion, smaller particle size, and higher density than of undoped samples [6,8,9]. Due to the large difference between the ionic radii of the dopant and the substituted cations, doping with La^3+^ is energetically unfavorable [12]. CaFe_2_O_4_ has attracted significant attention in various applications due to its excellent magnetic properties, narrow band gap and, consequently, easy absorption of photons in the visible light range [28]. Though, to our knowledge, no studies report the synthesis and characterization of Ca doped CoFe_2_O_4_.

The properties of ferrite nanoparticles are greatly sensitive to their elemental composition, crystal structure, dopants, synthesis method, and preparation conditions [1]. Various methods were developed for the preparation of undoped CoFe_2_O_4_ and M-doped CoFe_2_O_4_, such as co-precipitation, sol-gel, thermal decomposition, microwave-assisted, laser pyrolysis, mechanical size reduction, gas phase synthesis, thermal hydrolysis, pulse laser deposition, microemulsion, solvothermal, sonochemical, mechanical–chemical processing, ball milling, hydrothermal synthesis, auto-combustion, etc., with each method having their advantages and shortcomings [4,8,9,21,22,23,24]. Of these, the chemical methods are susceptible to nanoparticle agglomeration; self-agglomeration is one of the key impediments in the obtaining of magnetic nanoparticles and can be diminished by their embedding into inorganic or organic matrices or by using surfactants to cover the nanoparticle surface [11].

The sol-gel route has the advantages of simplicity, flexibility, low cost, good control over the structure and properties, and production of nanosized ferrites with trustable and reproducible physical properties [11,21,22,23]. To stabilize and diminish nanoparticle agglomeration in order to obtain single-phase ferrites, the nanoparticles can be coated by a uniform and stable ultrathin layer or dispersed in a non-magnetic matrix. The sol-gel method involves mixing reactants with tetraethylorthosilicate (TEOS), forming strong networks with moderate reactivity, allowing the incorporation of various organic and inorganic molecules and short gelation time [21,22,23]. In this regard, forming an inactive SiO_2_ coating on the surface of oxide systems could prevent their agglomeration and improve their chemical stability, with the SiO_2_ acting as a physical barrier that control the attraction between the magnetic nanoparticles [21,22,23,29,30]. Moreover, embedding magnetic ferrites into the SiO_2_ matrix gained considerable attention due to the possibility of controlling the particle size and minimizing the surface roughness and spin disorder, thus enhancing the magnetic properties of the obtained nanocomposites [22,23]. Additionally, the non-magnetic SiO_2_ matrix possesses a high surface area and does not affect the magnetic behavior or electric properties of the CoFe_2_O_4_ nanoparticles due to its lower dielectric constant [29]. 

In the present work, we investigate the changes in the structural, morphological, surface, and magnetic properties of undoped and doped CoFe_2_O_4_ with transition monovalent (Ag^+^, Ag_0.1_Co_0.95_Fe_2_O_4_; Na^+^, Na_0.1_Co_0.95_Fe_2_O_4_), divalent (Ca^2+^, Ca_0.1_Co_0.9_Fe_2_O_4_; Cd, Cd_0.1_Co_0.9_Fe_2_O_4_) and trivalent (La^3+^, La_0.1_CoFe_1.9_O_4_) metal ions embedded in the SiO_2_ matrix obtained through the sol-gel route, followed by calcination at various temperatures. 

The novelties of this paper consist of the following:

(i) A study of the influence of the SiO_2_ matrix and dopant ion on forming metallic succinates and their decomposition into ferrites embedded in the SiO_2_ matrix.
(ii) A comparative study of the impact of monovalent, divalent, and trivalent ion doping of CoFe_2_O_4_ embedded in the SiO_2_ matrix on the morphological, magnetic and structural properties in order to find new strategies to increase their potential for existing and new possible applications.
(iii) Filling the gap in the existing literature on the effect of Na^+^, Ag^+^, Cd^2+^, La^3+^, and Ca^2+^ ion doping on the physicochemical properties of CoFe_2_O_4_ embedded in the SiO_2_ matrix; the embedding of CoFe_2_O_4_ nanoparticles in the non-toxic, inert SiO_2_ matrix via the sol-gel method allows the control of the particle growth, reduces the nanoparticle agglomeration and enhances the chemical stability and magnetic guidance [11].
(iv) The elucidation of the thermal behavior under isothermal and non-isothermal conditions and the formation kinetics of undoped and doped CoFe_2_O_4_.
(v) Obtaining single crystalline ferrites at low calcination temperatures (400 and 700 °C) by doping CoFe_2_O_4_ with mono-, di- and trivalent ions.

## 2. Results and Discussion

### 2.1. Thermal Stability of CoFe_2_O_4_ and Role of Doping Metals

**Figure 1:**
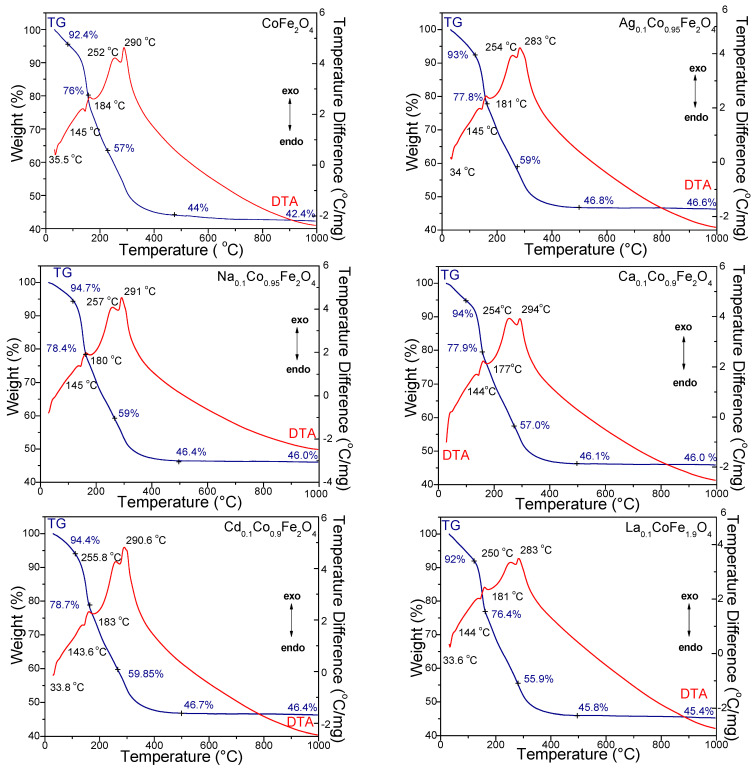
TG and DTA of CoFe_2_O_4_, Ag_0.1_Co_0.95_Fe_2_O_4_, Na_0.1_Co_0.95_Fe_2_O_4_, Ca_0.1_Co_0.9_Fe_2_O_4_, Cd_0.1_Co_0.9_Fe_2_O_4_ and La_0.1_CoFe_1.9_O_4_ gels heated at 40 °C.

Figure 1 shows the TG and DTA curves for the gels dried at 40 °C. The DTA curves (Figure 1) of the gels dried at 40 °C show the following three processes: (i) the loss of residual water (physically adsorbed water and moisture) indicated by the endothermic peak with the maximum at 33.6–35.5 °C, with a mass loss of 5.3–7% between 25 and 110 °C; (ii) the formation of metal succinates indicated by two endothermic effects at 143.6–145 °C (formation of Co, Ag, Na, Ca, and Cd succinates) and 177–184 °C (formation of Fe and La succinates) with a mass loss of 14.4–16.3% and (iii) the decomposition of metal succinates to metal oxides and the formation of ferrites shown by two exothermic effects at 250–257 °C (mass loss of 18.8–20.9%), corresponding to the oxidative decomposition of the succinates to Ag, Na, Co, Ca, and Cd oxides and at 283–294 °C (mass loss of 10.1–13.2%), consistent with the formation of Fe and La oxides [21,22,29,30]. The two-stage (144–145 °C and 177–184 °C) evolution of the redox reaction between the metal nitrates and 1,4-butanediol (1,4-BD) is due to the free aqueous, stronger acid [Fe(H_2_O)_6_]^3+^ ion than [Co(H_2_O)_6_]^2+^ ion [21]. As a result, the Fe succinate is formed at higher temperatures [21]. The corresponding mass loss on the TG curve is due to the loss of crystallization water from the nitrates and volatile products (H_2_O, NO_2_), resulting in the redox reaction [21]. An additional mass loss (0.1–1.6%) can also be observed on some TG curves between 900 and 1000 °C. This effect is the most visible in the undoped CoFe_2_O_4_. The highest total mass loss is observed for CoFe_2_O_4_ (57.6%), while in the doped CoFe_2_O_4_, the total mass loss is slightly lower (53.4–54.6%). The SiO_2_ matrix undergoes various transformations during the thermal process, which makes the processes’ delimitation ascribed to the formation and decomposition of succinate precursors difficult [21,22,23,29,30].

**Figure 2:**
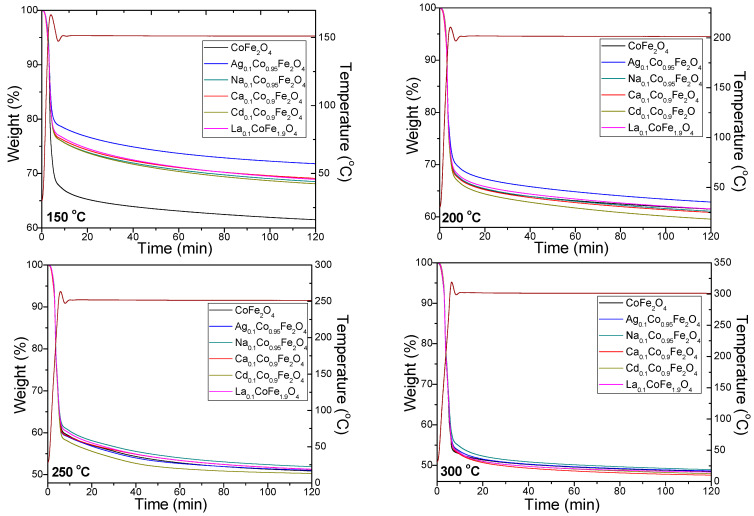
TG isotherms of CoFe_2_O_4_, Ag_0.1_Co_0.95_Fe_2_O_4_, Na_0.1_Co_0.95_Fe_2_O_4_, Ca_0.1_Co_0.9_Fe_2_O_4_, Cd_0.1_Co_0.9_Fe_2_O_4_ and La_0.1_CoFe_1.9_O_4_, gels.

The decomposition of metal succinates under isothermal conditions (150, 200, 250, and 300 °C) is presented in Figure 2. In all cases, a rapid mass loss in the first 10 min, followed by a slow mass loss of up to 120 min, is observed. The mass loss is lower at 150 and 200 °C due to the incomplete formation of metal succinates and is comparable at 250 and 300 °C, confirming the formation of ferrites around 250 °C. Ag_0.1_Co_0.95_Fe_2_O_4_ presents the lowest mass loss on the 150 and 200 °C isotherms, while Na_0.1_Co_0.95_Fe_2_O_4_ presents the lowest mass loss on the 250 and 300 °C isotherms. The highest mass loss appears on the 150 °C isotherm for CoFe_2_O_4_, on the 200 °C and 250 °C isotherms for Cd_0.1_Co_0.9_Fe_2_O_4_, and the 300 °C isotherm for Ca_0.1_Co_0.9_Fe_2_O_4_.

### 2.2. Kinetics of Doped and Undoped CoFe_2_O_4_ Formation

The rate constant (*k*) was calculated using the isotherms recorded at 150, 200, 250 and 300 °C according to the following first-order kinetic equation, Equation (1):

(1) $$\frac{dx}{dt}=k{\left(x_{o}-x\right)}^{2/3}$$

where *dx/dt* is the reaction rate, *x_o_* is the initial mass (mg), *x* is the mass (mg) at time *t*, *t* is the time and *k* is the rate constant defined as *k = A e^(−E/RT)^*, where *A* is the pre-exponential factor, *E* is the activation energy, *R* is the ideal gas constant, and *T* is the temperature [31]. The integration of Equation (1) leads to Equation (2) as follows:

(2) $${\left(x_{o}-x\right)}^{1/3}={\left(x_{o}\right)}^{1/3}-kt$$

Each isotherm’s rate constant (*k*) value was computed using ten values in the 0–10 min range, where the highest mass loss occurs. A higher *k* indicates a faster reaction [31,32]. The *k* value increases with the increase in the calcination temperature. At 150 and 200 °C, the average k values of the doped ferrites are lower than that of the undoped CoFe_2_O_4_, except for Ca_0.1_Co_0.9_Fe_2_O_4_. At 250 and 300 °C, the average k value is higher for Ag_0.1_Co_0.95_Fe_2_O_4_, Ca_0.1_Co_0.9_Fe_2_O_4_, and La_0.1_CoFe_1.9_O_4_, and lower for Na_0.1_Co_0.95_Fe_2_O_4_ and Cd_0.1_Co_0.9_Fe_2_O_4_ compared with the average k value of the undoped CoFe_2_O_4_ (Table 1).

**Figure 3:**
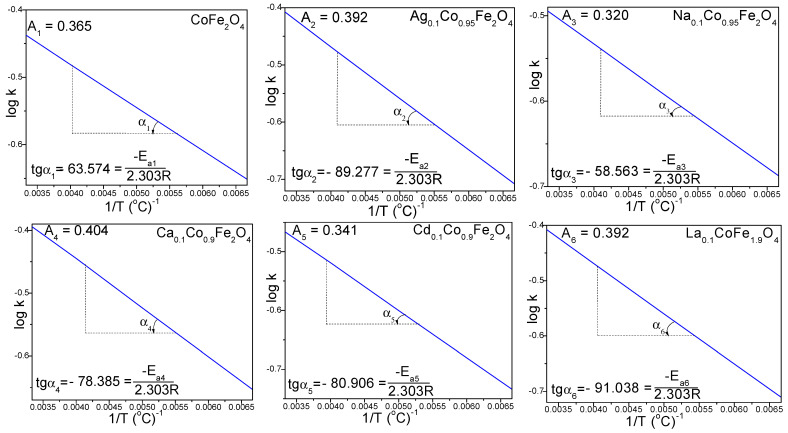
The log *k* vs. 1/*T* plots and pre-exponential factors (*A*) of CoFe_2_O_4_, Ag_0.1_Co_0.95_Fe_2_O_4_, Na_0.1_Co_0.95_Fe_2_O_4_, Ca_0.1_Co_0.9_Fe_2_O_4_, Cd_0.1_Co_0.9_Fe_2_O_4_ and La_0.1_CoFe_1.9_O_4_ gels heated at 150, 200, 250 and 300 °C.

The activation energy (*E_a_*) and the pre-exponential factor (*A*) for the formation of doped and undoped CoFe_2_O_4_ were calculated by plotting the logarithm of the rate constant (*k*) versus the inverse temperature, log *k* vs. 1/*T* (Figure 3). The activation energy (*E_a_*) was calculated according to the Arrhenius equation (Equation (3)) [32] as follows:

(3) $$\log k=\log A-\frac{E_{a}}{2.303R}\cdot \frac{1}{T}$$

where *A* is the pre-exponential factor, *E_a_* is the activation energy (J/mol), *R* is the ideal gas constant (8.314 J/mol·K) and *T* is the temperature (*K*). The *E_a_* value of the undoped CoFe_2_O_4_ (1.217 kJ/mol) increases by doping with Ag^+^, Ca^2+^, Cd^2+^ and La^3+^, and decreases by doping with Na^+^ (Table 1).

### 2.3. FT-IR Spectroscopy

**Figure 4:**
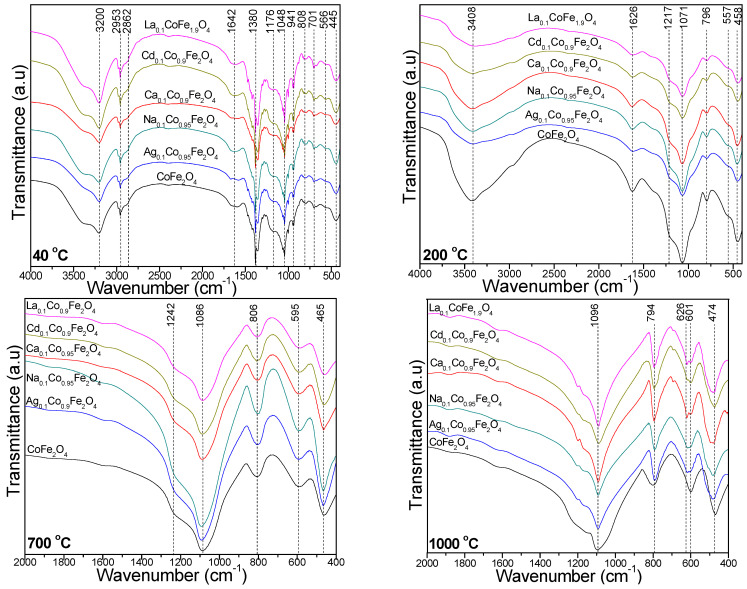
FT-IR spectra of gels heated at 40 and 200 °C and calcined at 700 and 1000 °C.

The FT-IR spectra (Figure 4) of the gels heated at 40 °C show an intense band around 1380 cm^−1^, which is characteristic to nitrate groups, two bands at 2953 and 2862 cm^−1^, which are specific to C–H bond vibrations and a band at 3200 cm^−1^, which is attributed to intermolecular hydrogen bonds in 1,4-BD [22,23]. These bands are not remarked in the spectra of the gels heated at 200 °C, confirming the decomposition of metal nitrates and the formation of the metal succinates, respectively.

The vibrations of the OH groups in 1,4-BD and the adsorbed molecular water appear at 1642 cm^−1^, while the band at 950–944 cm^−1^ is attributed to the deformation vibration of Si-OH that occurs during the hydrolysis of the –Si(OCH_2_CH_3_)_4_ groups in TEOS [21,22,23]. The band at 808 cm^−1^ is attributed to the stretching vibration of the Si–O chains in the SiO_4_ tetrahedron, the band at 1048 cm^−1^ is attributed to the stretching vibration of the Si–O–Si bonds, while the shoulder at 1176 cm^−1^ is attributed to the stretching vibration of the Si–O bonds in the SiO_2_ matrix [22,23]. As shown in the XRD data, in the samples calcined at low temperatures (400, 700 °C) the SiO_2_ is amorphous, while in the samples calcined at 1000 °C, the SiO_2_ (quartz and cristobalite) is crystalline. In general, macro-sized silica is crystalline, and nano-sized silica is amorphous.

At 200 °C, the band around 1626 cm^−1^ is attributed to the vibration of the COO^−^ groups, indicating the formation of a chelated complex through the coordination of succinates by metal ions. The disappearance of this band in the FT-IR spectra of the samples calcined at 700 and 1000 °C suggests that the formed cobalt ferrite nanoparticles have no residual organic compounds. The band at 3408 cm^−1^ is attributed to O–H stretching and intermolecular hydrogen bonds in metal succinates [22,23]. The samples heated at 40 and 200 °C show that the absorption band around 560 cm^−1^ is assigned to the stretching vibrations of tetrahedral M–O bonds and cyclic Si–O–Si structures, while the band at around 450 cm^−1^ is attributed to the octahedral M–O and Si–O bond vibrations. The presence of the SiO_2_ matrix is indicated by the symmetric stretching and bending vibration of the Si–O–Si chains at around 800 cm^−1^ and the stretching vibration of the Si–O–Si bonds at around 1071 cm^−1^, with a shoulder at around 1217 cm^−1^ [22,23]. The FT-IR spectra of the gels calcined at 700 and 1000 °C (Figure 4) show the specific bands of the SiO_2_ matrix as follows: Si–O–Si bond stretching vibration (1086–1096 cm^−1^ and the shoulder at 1242 cm^−1^), Si–O chain vibration in the SiO_4_ tetrahedron (794–806 cm^−1^), Si–O bond vibration (465–474 cm^−1^) and Si–O–Si cyclic structure vibration (595–601 cm^−1^) [22,23]. At 1000 °C, the supplementary band at 626 cm^−1^ is attributed to cristobalite. This band does not appear for the CoFe_2_O_4_ calcined at 1000 °C, supporting the obtained XRD results. The stretching vibrations of the Ag–O, Na–O, Ca–O, Co–O, Cd–O, and La–O bonds are slightly shifted toward lower wavenumbers (595–601 cm^−1^), while the Fe–O bond vibration is shifted toward slightly higher wavenumbers (465–474 cm^−1^) [21,22,23,24]. The shift of these bands’ positions in samples with different doping cations and different calcination temperatures is a consequence of the distortion of the lattice due to different M–O distances [33]. The bands at 794–806 cm^−1^ are attributed to the O–Fe–O and Fe–OH bond vibrations, while the band at 465–474 cm^−1^ is ascribed to the Fe–O linkage [24]. 

### 2.4. X-ray Diffraction of Undoped and Doped CoFe_2_O_4_

As the oxidic phases at low temperatures are poorly crystalline or amorphous, the desired surface properties and crystallinity can be achieved by tailoring the calcination conditions. In addition, the reactivity of the amorphous SiO_2_ allows its participation in various chemical transformations.

**Figure 5:**
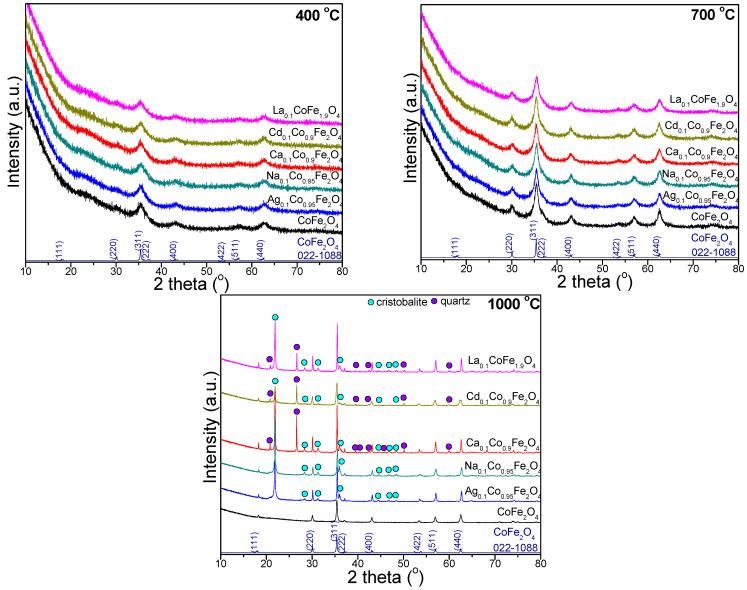
XRD patterns of gels calcined at 400, 700 and 1000 °C.

The XRD patterns of the gels calcined at 400, 700, and 1000 °C are presented in Figure 5. At 400 and 700 °C, the diffraction peaks matching with the reflection planes of (220), (311), (222), (400), (422), (511), and (440) confirm the presence of the pure, low-crystallized CoFe_2_O_4_ (JCPDS #00-022-1086) phase with a cubic spinel structure (space group *Fd3m*) [22,23,24]. At low temperatures (400 and 700 °C), doping ions did not produce any secondary impurity-associated reflections, and the spinel crystal structure of the produced gels was maintained. The absence of secondary phases points toward the successful insertion of doping ions. The broad peak at 2θ = 20–40° reveals the low crystallization of gels calcined at 400 and 700 °C. At 1000 °C, the undoped ferrite displays the single, well-crystallized CoFe_2_O_4_ phase, which is accompanied by cristobalite (JCPDS #89-3434) for the Na- and Ag-doped ferrites and by cristobalite and quartz (JCPDS #85-0457) for the Ca-, La- and Cd-doped ferrites. Calcination also led to a slight shift in the 2θ peak position, small changes in the peak width, and higher crystallite sizes [22,23]. The Cd_0.1_Co_0.9_Fe_2_O_4_ gel displays the lowest intensity diffraction peaks, indicating the lowest crystallization compared to the other gels. The increase in the diffraction peaks’ intensity with the calcination temperature indicates the increase in the crystallinity degree and crystallite size [22,23]. The presence of La^3+^ ions did not generate any secondary impurity-related reflections, and the spinel crystal structure of the CoFe_2_O_4_ was maintained [6]. Oppositely, Mansour et al. reported the appearance of LaFeO_3_ as a secondary phase due to the diffusion of some La^3+^ ions to the grain boundaries that react with Fe to form LaFeO_3_ [8]. The XRD patterns are influenced not only by the calcination temperature and doping ions but also by the crystallite size, lattice strain, and defects [22].

The structural parameters, namely, the crystallite size (D_XRD_), degree of crystallinity (DC), lattice parameter (a), unit cell volume (V), distance between the magnetic ions and the hopping length in the A (d_A_) and B (d_B_) sites, physical density (d_p_), X-ray density (d_XRD_) and porosity (P), of the gels calcined at 400, 700 and 1000 °C determined by XRD are presented in Table 2. The D_XRD_ increases with the calcination temperature since at high temperatures (1000 °C), the crystallite agglomeration without subsequent recrystallization led to the formation of a single crystal rather than a polycrystal structure [22]. Mariosi et al. reported an increase in the crystallite size from 4.5 nm to 4.9 nm for CoLa_0.05_Fe_1.95_O_4_ for a higher La^3+^ content (CoLa_0.1_Fe_1.9_O_4_) [6].

The DC is the ratio of the area of crystalline peaks over the total area of the diffractogram. A possible reason for the slight reduction in D_XRD_ by Cd^2+^ doping could be the local temperature increase to release the latent energy on the surface. This process leads to a strike in the crystal growth and lowers the concentration of ferrites in the vicinity [3]. The D_XRD_ increases for the doped ferrites, except in the case of Cd^2+^ doping (Table 2). The observed expansion of the unit cell and the high structural distortion of the doped CoFe_2_O_4_ compared to the undoped CoFe_2_O_4_ are attributed to the difference in the ionic radii of the host and dopant ions, as well as to the change in the cation distribution that occurs due to the Ag^+^, Na^+^, Ca^2+^, Cd^2+^, and La^3+^ doping in the spinel structure [1,4,6,27]. A possible explanation for the largest unit cell value of La_0.1_CoFe_1.9_O_4_ is the much larger ionic radius of La^3+^ (1.216 Å) than of Fe^3+^ (0.65 Å), with the unit cell expansion via doping with La^3+^ taking place to compensate for the crystal deformation; accordingly, Shang et al. stated that the replacement of Fe^3+^ ions by La^3+^ ions result in a higher potential barrier for the formation of a spinel ferrite crystal structure [9]. The lattice parameter of the undoped and doped CoFe_2_O_4_ gels increases with the calcination temperature, which is also ascribed to the expansion of the unit cell [27]. A possible explanation for the difference between the theoretical and experimental values could be the assumption that the ions are rigid hard spheres [22]. The obtained results and the absence of supplementary phases in the XRD patterns indicate that the doping ions are incorporated into the CoFe_2_O_4_ structure [5,6]. The increase in the molecular weight is more significant than the increase in the V (Table 2); however, the molecular weight is more influenced by the increase in the V [22]. The decrease in the unit cell volume is expected with the introduction of smaller-sized monovalent (Ag^+^, Na^+^) ions in the crystal lattice. The d_A_ and d_B_ of the gels calcined at 700 and 1000 °C are higher for the doped CoFe_2_O_4_ than the undoped CoFe_2_O_4_ and display a decreasing trend for the monovalent dopant (Ag^+^, Na^+^) ion and an increasing trend for the trivalent dopant (La^3+^) ion (Table 2). The lower value of the d_p_ (Table 2) of the undoped CoFe_2_O_4_ compared to the doped CoFe_2_O_4_ could be attributed to the pore formation through the synthesis processes [22]. The variation of the d_p_ caused by small fluctuations in the lattice constant is probably due to the changes in the cation distribution among the A and B sites. The P (Table 2) of the doped CoFe_2_O_4_ is lower than that of the undoped CoFe_2_O_4_. Additionally, the rapid densification during the calcination and the growth of irregular shape grains decrease the porosity at higher calcination temperatures. The decrease in the P with the increase in the d_p_ may result from the different grain sizes [22]. In conclusion, the increase in the D_XRD_, DC, a, V, d_A_, d_B_, and d_p_, and the decrease in the d_XRD_ and P at higher calcination temperatures are observed. 

### 2.5. Elemental Composition of Undoped and Doped CoFe_2_O_4_

To investigate the elemental composition and verify the stoichiometric amount of each element in the undoped and doped CoFe_2_O_4_, the M/Co/Fe molar ratio was determined using inductively coupled plasma optical emission spectrometry (ICP-OES) after microwave digestion (Table 2). The best fit between the experimental and theoretical data is observed for the gels calcined at 1000 °C.

### 2.6. Morphology and Surface Parameters of Undoped and Doped CoFe_2_O_4_

**Figure 6:**
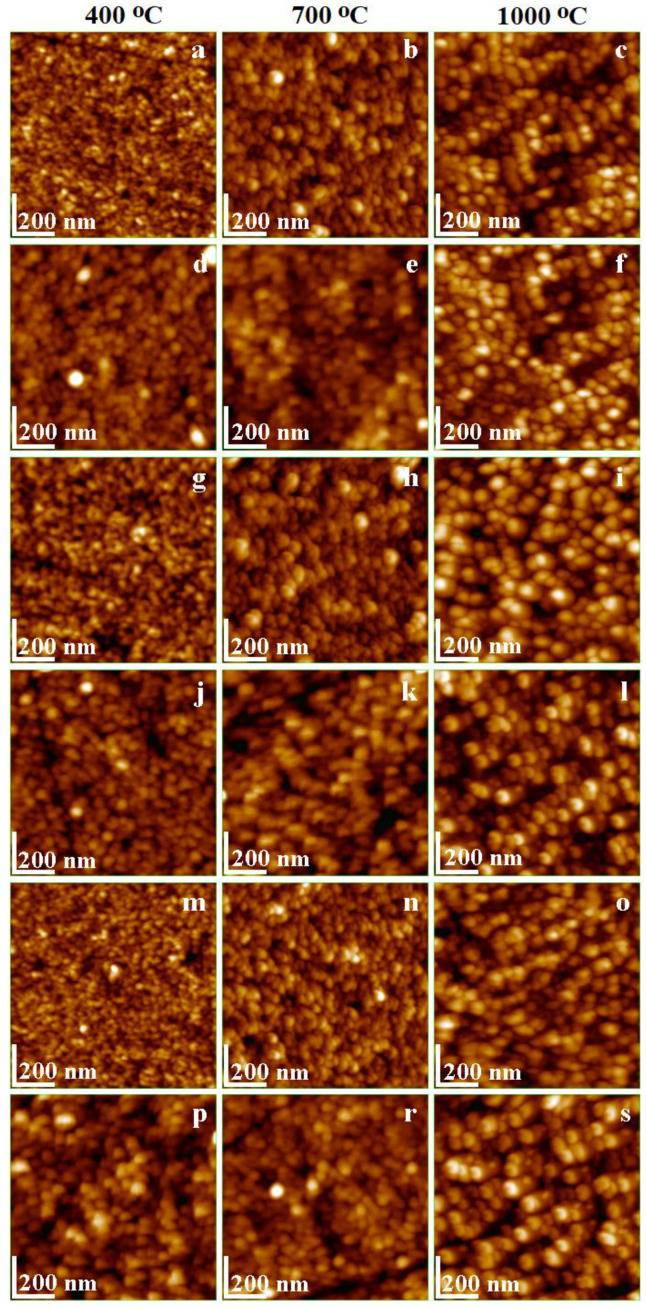
AFM topographic images of CoFe_2_O_4_ (**a**–**c**), Ag_0.1_Co_0.95_Fe_2_O_4_ (**d**–**f**), Na_0.1_Co_0.95_Fe_2_O_4_ (**g**–**i**), Ca_0.1_Co_0.9_Fe_2_O_4_ (**j**–**l**), Cd_0.1_Co_0.9_Fe_2_O_4_ (**m**–**o**) and La_0.1_CoFe_1.9_O_4_ (**p**–**s**) gels calcined at 400, 700 and 1000 °C.

The thermal treatment concomitantly enables the crystalline phase formation and grain growth with a relative coalescence between the particles. The coalescence can be attributed to the physical attraction forces between the small particles or the bonding bridges between the particles, especially at high calcination temperatures [34,35]. As the microscopic examination of gels failed due to the agglomeration of small particles into clusters, the powders were dispersed into deionized water under intense stirring to break up the powder clusters and release the free particles into dispersion. The water dispersion also prevents the particles’ re-agglomeration and allows their transfer onto a solid substrate as a thin film through adsorption [36,37]. This method is usually used to prepare thin films of noble metal nanoparticles directly from the mother solution [38,39], but it was also successfully used for the Ni- and Mn-doped ferrites [29,30]. The obtained thin films were subjected to atomic force microscopy (AFM) (Figure 6). Mansour et al. suggested a coalescence phenomenon, resulting in large particle size of the obtained La-doped CoFe_2_O_4_ nanoparticles [8].

The undoped CoFe_2_O_4_, after calcination at 400 °C, displays small spherical particles of around 25 nm (Figure 6a), which increase to 30 nm with the calcination temperature at 700 °C (Figure 6b), and to 40 nm at 1000 °C (Figure 6c). The polycrystalline particles formed at 400 °C consist mainly of low-crystalized CoFe_2_O_4_ mixed with amorphous material. At 700 °C, the growth of CoFe_2_O_4_ crystallites and the presence of amorphous matter are remarked. For the gels calcined at 1000 °C, the crystallite size estimated via XRD (37.2 nm) is comparable with the particle size observed using AFM (around 40 nm). The undoped CoFe_2_O_4_ calcined at 1000 °C is mono-crystalline, as indicated by the spherical particle shape with slightly square corners (Figure 6c). These results are in good agreement with the data in the literature [40,41].

The Ag-doped cobalt ferrite calcined at 400 °C is low crystalline, with crystallites of 14 nm mixed with amorphous matter into small spherical particles of around 27 nm (Figure 6d). Increasing the calcination temperature to 700 °C leads to better-developed crystallites of 24.7 nm and increases the particle size to about 31 nm. The development of the crystalline phase and the reduction in the amorphous component determines a significant alteration of the particle shape, which becomes spheroidal (Figure 6e). The particle shape evolving tendency continues by calcination at 1000 °C, with an increase in size to 70 nm and a crystalline core of 65.1 nm (Figure 6f). The presence of secondary phases prevents the development of cubic shape features, which remains spherical, as reported by Prabagar et al. [42]. Mahajan et al. also reported a higher Ag-doped CoFe_2_O_4_ crystallite size than the undoped CoFe_2_O_4_ [43].

The Na-doped cobalt ferrite shows no significant modification of the crystallite size for the gels calcined at 400 °C and 700 °C (Table 2). Like the undoped CoFe_2_O_4_, the amorphous matter between the crystallites leads to slightly larger spherical particles of 24 nm at 400 °C, and 35 nm at 800 °C (Figure 6g,h). Surprisingly, after calcination at 1000 °C, well-structured particles with a 52 nm diameter and a ferrite core of 47.6 nm covered with some traces of cristobalite are remarked (Figure 6i). Due to the presence of the crystalline core, the particle shape becomes spheroidal. The observed size and shape are in good agreement with the data in the literature [44].

The Ca-doped cobalt ferrite, calcined at low temperatures, exhibits spherical particles of around 28 nm (Figure 6j), formed by a 14.6 nm ferrite crystallite core coated with amorphous material. Kumar and Kar also reported crystallites of 10 nm for this composition by calcination at 550 °C for 2 h [45]. Higher calcination temperature at 700 °C facilitates the crystal growth, leading to crystallites of 25.2 nm mixed with some amorphous matter, which generates particles of about 33 nm. Predominately spherical particles are accompanied by several right corners formed on the most representative particles (Figure 6k). Calcination at 1000 °C generates well-formed Ca_0.1_Co_0.9_Fe_2_O_4_ crystallites of around 73.7 nm. Due to the traces of cristobalite and quartz crystalline phases, the crystallites become the core of the particles of about 75 nm (Figure 6l). The particle has cubic shapes with rounded edges, which is in good agreement with the data in the literature [46]. Only a few blunted cristobalite and quartz particles are observed around ferrites. 

The Cd-doped cobalt ferrite gels calcined at 400 and 700 °C present small spherical particles of around 20 and 28 nm, respectively, containing small ferritic crystallites and amorphous material (Figure 6m,n). The heterogeneous nanostructure of Cd_0.1_CoFe_1.9_O_4_ was also evidenced by Shakil et al. [47]. The calcination at 1000 °C leads to particles of around 40 nm (Figure 6o) having a ferrite core of 35 nm, which is in good agreement with the less developed XRD peaks. Particle sizes of 30–50 nm were previously reported for a similar composition [3]. 

The La-doped cobalt ferrite has a significant influence on the particle size and shape. The calcination at 400 °C results in spherical particles of around 30 nm containing crystallites of 15.5 nm mixed with amorphous material (Figure 6p). A possible explanation could be the higher value of the La atomic radius (2.50 Å) compared to that of Fe (1.26 Å) [48]. This effect is enhanced by calcination at 700 °C, resulting in particles of 38 nm with a crystalline core of 26.1 nm (Figure 6r), covered with amorphous material. 

Figure 6n shows several bigger particles that might indicate that the crystallization process is in progress, but most particles do not have enough time to reach a larger size. By calcination at 1000 °C, particles of around 90 nm, with a ferrite crystalline core of 81.8 nm covered by traces of cristobalite and quartz (Figure 6s), are formed. The spherical particle features a specific aspect derived from a cubic crystallite core, which is in good agreement with the data in the literature [49,50]. Some small particles of about 40–50 nm belong to the secondary phases. The particle adsorption onto the solid substrate develops thin films with specific topographic characteristics, which depend on the morphological aspects correlated with their density on the surface [51,52].

**Figure 7:**
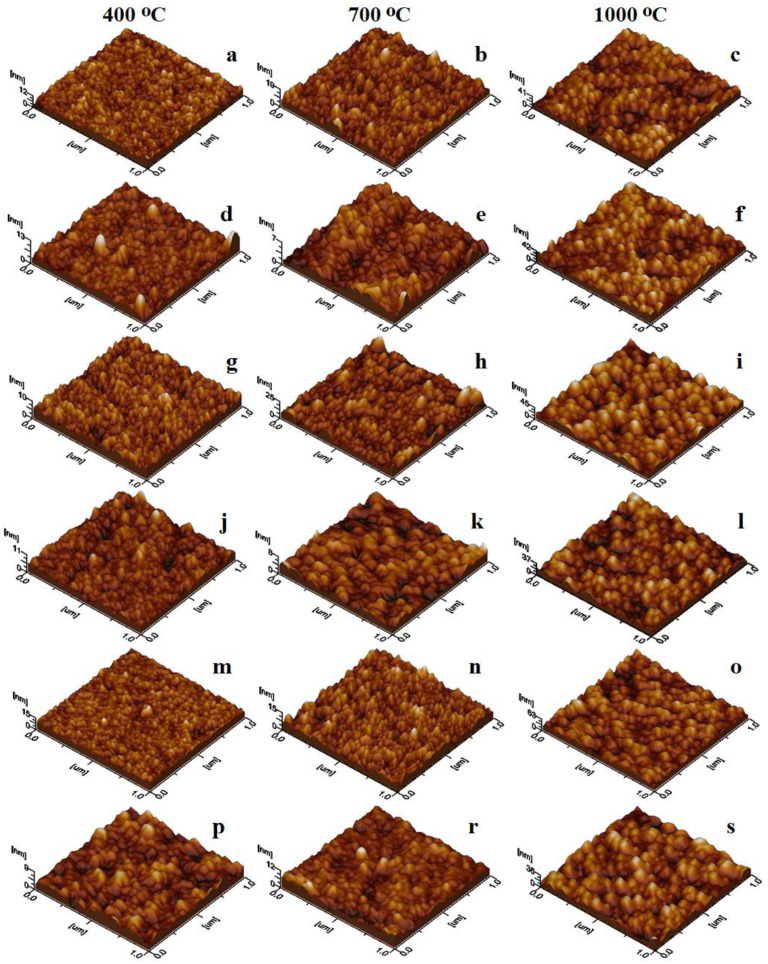
Tridimensional profiles of CoFe_2_O_4_ (**a**–**c**), Ag_0.1_Co_0.95_Fe_2_O_4_ (**d**–**f**), Na_0.1_Co_0.95_Fe_2_O_4_ (**g**–**i**), Ca_0.1_Co_0.9_Fe_2_O_4_ (**j**–**l**), Cd_0.1_Co_0.9_Fe_2_O_4_ (**m**–**o**) and La_0.1_CoFe_1.9_O_4_ (**p**–**s**) gels calcined at 400, 700 and 1000 °C.

The tridimensional profiles of the undoped CoFe_2_O_4_ and the Ca-, Na- and Cd-doped CoFe_2_O_4_ reveal that the particles resulting after calcination at 400 °C build uniform films of well-individualized nanoparticles (Figure 7a,d,p). Interestingly, the doping with Ag and La leads to a slightly irregular film due to the occurrence of local heights (Figure 7g,m), probably due to the irregular adsorption generated by local influences related to the bigger crystallite sizes. The particles obtained by calcination at 700 °C generally display a complex topography of the thin film with randomly spotted bigger particles, which may contain a more developed crystalline core for all the doped CoFe_2_O_4_ gels, except for Cd (Figure 7e,h,k,n). The undoped ferrite and Cd_0.1_Co_0.9_Fe_2_O_4_ calcined at 700 °C form uniform and smooth films (Figure 7b,r). 

The surface roughness (Rg) values are presented in Table 3. The particles formed by calcination at 1000 °C are well individualized and present crystalline topographic aspects and form relatively uniform thin films with no signs of coalescence tendency (Figure 7c,f,i,l,o,s). The observed topographic aspects may be helpful in the further processing of the obtained particles as the main ingredient for dedicated thin film preparation.

The AFM investigation allows for the effective area of topographic features at a precise scanned area to be measured. Hence, the thin films obtained through adsorption from aqueous dispersion are uniform and compact (Figure 6), allowing the measurement of powder surface area. The particle number and diameter influence the variation of the obtained values (Table 3) since a large number of bigger particles leads to a large powder area, while a small number of particles with a small diameter leads to a small powder area [22,23]. Therefore, low calcination temperatures generate thin films with a small surface area by spreading the secondary phases among ferrite particles. The well-crystallized ferrites obtained after calcination at 1000 °C form thin films with a significantly larger powder surface area. 

**Figure 8:**
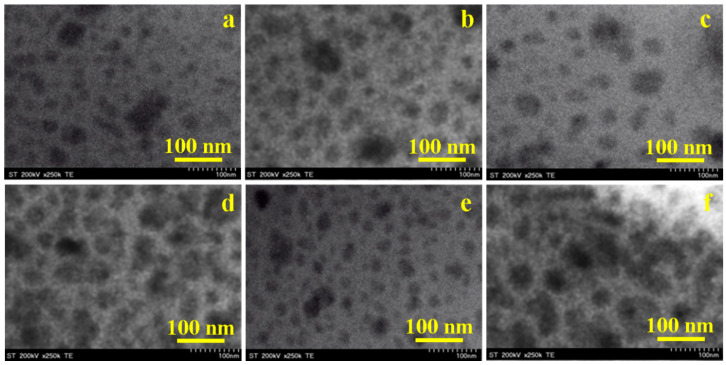
TEM images of (**a**) CoFe_2_O_4_, (**b**) Ag_0.1_Co_0.95_Fe_2_O_4_, (**c**) Na_0.1_Co_0.95_Fe_2_O_4_, (**d**) Ca_0.1_Co_0.9_Fe_2_O_4_, (**e**) Cd_0.1_Co_0.9_Fe_2_O_4_ and (**f**) La_0.1_CoFe_1.9_O_4_ gels calcined at 1000 °C.

Since the AFM topographic images reveal the exterior aspect of the particles, in order to obtain information on the internal structure of the particles, the transmission electron microscopy (TEM) images were recorded on the gels calcined at 1000 °C (Figure 8), considering that the ferrite crystallites are better developed at this temperature. The crystallites appear in dark grey shades surrounded by a lighter gray hollow, indicating a dense ferrite core covered by a thin layer of SiO_2_. Some particles are associated in clusters of about 90–100 nm, but these clusters are not observed in the AFM images due to their sedimentation in the aqueous dispersion before transferring the nanoparticles onto the glass slide.

The undoped CoFe_2_O_4_ (Figure 8a) displays mainly fine particles of about 38 nm in diameter, which is in agreement with the AFM, but slightly bigger than the average value estimated by the XRD, probably due to the presence of the cristobalite and quartz exterior layer. A particle cluster of about 95 nm is observed on the central side of Figure 8a. Small and homogenously distributed particles result when the nucleation rate exceeds the growth rate, but the small particles tend to agglomerate into bigger structures. A possible explanation for the agglomeration tendency of small particles could be the interaction between the magnetic ions, van der Waals forces at the particle surface, and interfacial surface tensions [21,22,29,53,54]. The volume expansion and the internal energy produced during calcination may also lead to particle growth. The SiO_2_ matrix reduces the number of particles that interact with each other and, thus, reduces particle agglomeration [10,11,18,42,53,54].

Ag_0.1_Co_0.95_Fe_2_O_4_ presents spherical particles of about 67 nm (Figure 8b), which is very close to the value observed by the AFM. The dark core of the particles observed in the TEM images is very close to the average crystallite size estimated via XRD, while the light hollow surrounding the dark core suggests the ferrite coating by a thin layer of cristobalite and quartz, a fact also sustained by the spherical particle shape. The TEM images of Na_0.1_Co_0.95_Fe_2_O_4_ (Figure 8c) show a less dense distribution of spherical particles with a diameter of about 50 nm, which is in good accordance with the AFM. The intense gray shade indicates the presence of a 47.6 nm ferrite core inside the particles, which is in accordance with the crystallite diameter determined by the XRD data. Oppositely, the TEM image shows a compact structure of well-developed Ca_0.1_Co_0.9_Fe_2_O_4_ particles with a diameter of about 76 nm (Figure 8d). The particle size is slightly bigger than that observed by the AFM. The dark core corresponds to the ferrite crystallite evidenced by XRD, while the lighter halo on the exterior is attributed to the cristobalite and quartz layer. Figure 8e reveals a compact and uniform package of fine Cd_0.1_Co_0.9_Fe_2_O_4_ spherical particles of about 37 nm, which is in good agreement with the AFM. The core is darker due to the ferrite crystallite presence, and the lighter exterior shade corresponds to the crystalline SiO_2_ layer. La_0.1_CoFe_1.9_O_4_ presents big spherical particles of about 84 nm (Figure 8f), which is in good agreement with the AFM. The dark core corresponds to the ferrite, evidenced by XRD, and the outer halo to the cristobalite and quartz layer. The presence of some crystallite clusters indicates a local powder agglomeration. 

### 2.7. Magnetic Properties of Undoped and Doped CoFe_2_O_4_

**Figure 9:**
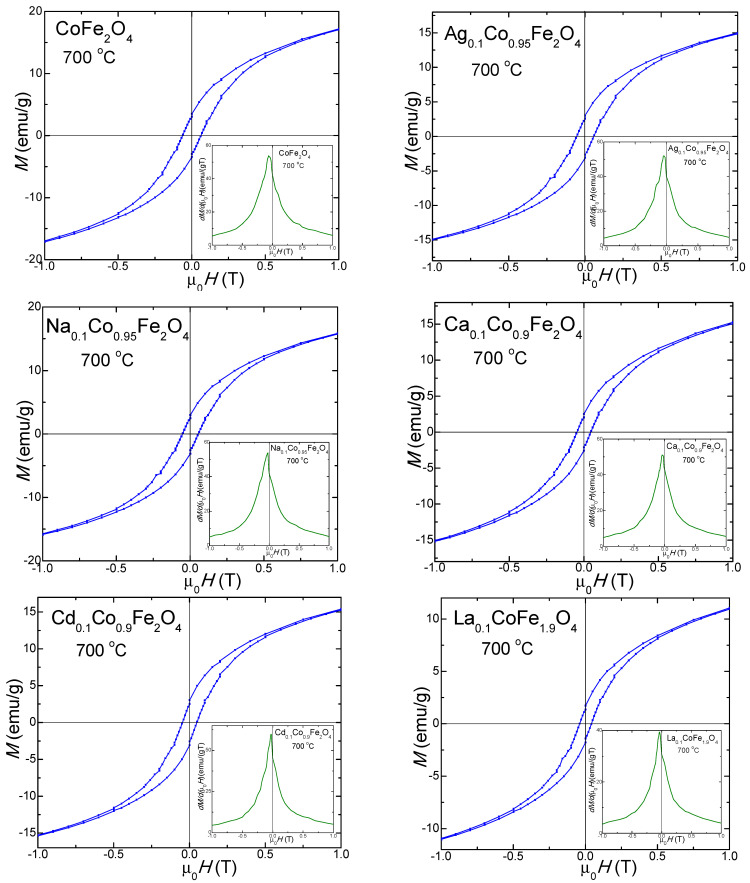
Magnetic hysteresis loops of gels calcined at 700 °C.

**Figure 10:**
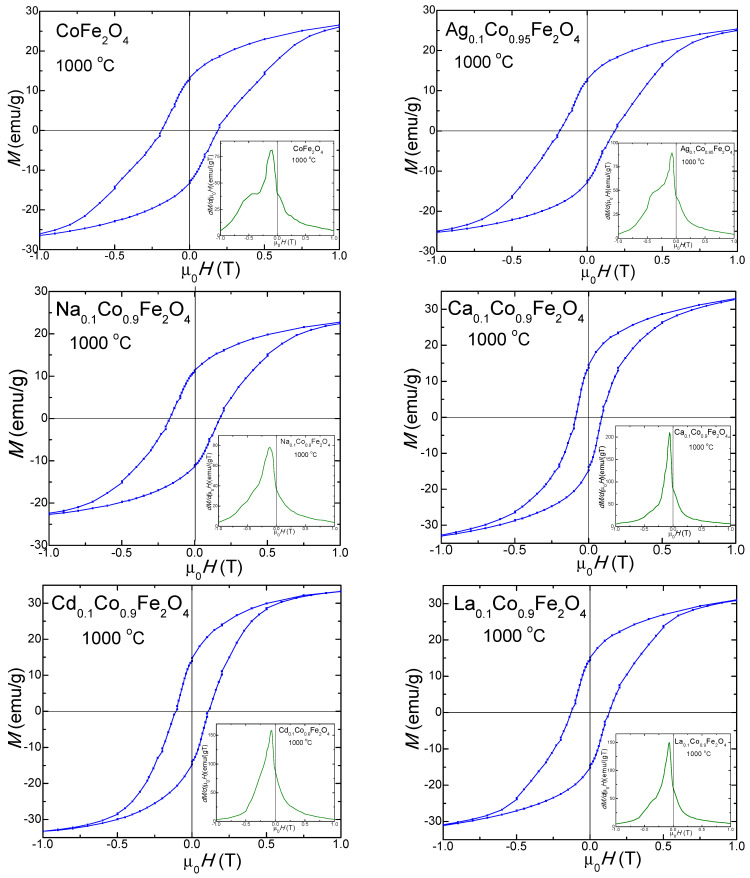
Magnetic hysteresis loops of gels calcined at 1000 °C.

The magnetic hysteresis loops, *M*(*µ_0_H*), and the magnetization first derivatives (*dM/d(μ_0_H*) of the gels calcined at 700 °C (Figure 9) and 1000 °C (Figure 10) indicate a typical ferromagnetic behavior. The derivative of the hysteresis loops (total susceptibility) is the local slope of the *M–H* curve. For the gels calcined at 700 °C, a single maximum in the *dM/d(μ_0_H)* vs. the *μ_0_H* curve, close to the coercive field, consistent with a single magnetic phase, is observed. These behaviors suggest crystalline samples with a single magnetic phase [21,22,23]. The magnetic hysteresis loops indicate moderate coercivity due to the coalescence of the particles accompanied by their magnetic coupling and improved magnetization. Although the magnetization first derivative *dM/d(μ_0_H)* of the undoped CoFe_2_O_4_ calcined at 1000 °C shows two maxima (a more intense and better differentiated maximum next to a less intense one), one on each side of the coercivity, these two magnetic phases are magnetically coupled inside of the particle along their magnetic moments [21,22,23]. The doping effect of the monovalent (Ag^+^ and Na^+^) ions supports the formation of the two magnetic phases (an intense peak and one as a shoulder merged with the other for Ag_0.1_Co_0.95_Fe_2_O_4_, and a broader maximum peak suggesting the merging of the two maxima, characteristic of the two magnetic phases for Na_0.1_Co_0.95_Fe_2_O_4_). Oppositely, the doping with the divalent (Ca^2+^ and Cd^2+^) and trivalent (La^3+^) ions improves the magnetic properties, leading to the formation of a single magnetic phase characterized by a single maximum, which is very intense and sharp on the *dM/d(μ_0_H)* vs. the *μ_0_H* curve.

Due to the change in the magnetocrystalline anisotropy or the particle sizes by doping CoFe_2_O_4_ with non-magnetic ions, the values of *M_S_*, remnant magnetization (*M_r_*), *H_C_*, magnetic moment per formula unit (*n*_B_), and *K* are higher than those of the undoped CoFe_2_O_4_, with few exceptions [1,4,26]. For the gels calcined at 1000 °C, the peak heights and their horizontal shifts are associated with the strength of the magnetic phases, with the broader peaks indicating a large particle size distribution accompanied by a large *H_C_* [21,22,23]. A significant increase in *H_C_* is observed on the hysteresis loops of the gels calcined at 1000 °C (they are much broader) compared to those at 700 °C. For the gels calcined at 1000 °C, the doping effect of the monovalent metals (Ag^+^, Na^+^) increases the already large *H_C_* of the undoped CoFe_2_O_4_, while the doping effect of the divalent (Ca^2+^, Cd^2+^) and trivalent (La^3+^) ions leads to a decrease in *H_C_*, which is observable on the hysteresis loops of the gels calcined at 1000 °C. 

The magnetic parameters *M_s_*, *M_R_*, *H_c_*, *n_B_* and *K* values determined using the hysteresis loops and *M(H)* curves are presented in Table 4. Generally, the *M_s_* for the spinel ferrites is dictated by the superexchange interactions between the A and B site cations. The *M_s_* decreases with the increase in the crystallite size due to the larger number of surface defects [55]. 

The highest *M_s_* value corresponds to CoFe_2_O_4_ calcined at 700 °C (30 emu/g), with the doping ions leading to lower *M_s_* values. For the sample calcined at 1000 °C, a slight increase in the undoped CoFe_2_O_4_ (31.5 emu/g) is remarked. The doping with monovalent (Ag^+^, Na^+^) ions results in lower *M_s_* values, while the doping with the divalent (Ca^2+^, Cd^2+^) and trivalent (La^3+^) ions, leads to higher *M_s_* values, with the highest *M_s_* values corresponding to Cd_0.1_Co_0.9_Fe_2_O_4_ (39.4 emu/g) for this bunch of gels. The surface effects at the nanoparticle surface include forming of a dead layer, which contains broken chemical bonds; deviations from the bulk cation distribution; randomly oriented magnetic moments; lattice defects and non-saturation effects, resulting in depreciated magnetic properties [21]. 

Cd_0.1_Co_0.9_Fe_2_O_4_ is a good candidate for various technological applications such as communication, data storage, and high-frequency inductors [1,4]. For both calcination temperatures, Ag_0.1_Co_0.95_Fe_2_O_4_ exhibits the lowest *M_S_* value. Previous studies also reported that the doping of diamagnetic Ag^+^ into the CoFe_2_O_4_ spinel structure substantially decreases the *M_S_* of CoFe_2_O_4_; a possible explanation for this is the high number of un-coordinated magnetic spins that are not able to align in the direction of the external magnetic field. Generally, the Ag doping enhances the nanoparticles’ antibacterial activities, suggesting that Ag_0.1_Co_0.95_Fe_2_O_4_ may be a potential candidate for antibacterial applications [4]. Moreover, considering the excellent electron conductivity of Ag, it is expected that Ag doping increases the catalytic activity of CoFe_2_O_4_ [25]. 

As La^3+^ is a non-magnetic ion, it does not participate in the exchange interactions with its nearest neighbor ion; thus, the superexchange interactions between the A and B sites’ cations are depreciated [55]. Above the single-domain critical size, the competition between the magneto-static energy and the domain-wall energy favors forming domain walls and splitting the single-domain particle into multi-domain particles [22]. Mariosi et al. reported that the *M_S_* of the undoped CoFe_2_O_4_ (44.6 emu/g) decreased to 29.0 emu/g for the first increase in the La^3+^ concentration (sample CoLa_0.025_Fe_1.975_O_4_); the possible mechanisms for the magnetic behavior of these nanoparticles are still widely discussed [6]. Moreover, a disorder in the crystal’s surface results in a lack of collinearity of magnetic moments; this effect is generally attributed to a single magnetic domain configuration [6]. When non-magnetic La^3+^ ions substitute Fe^3+^ ions, the content of Fe^3+^ ions at ferrite lattice sites is reduced, resulting in a decrease in the total magnetic moment and a weakening of the Fe^3+^–Fe^3+^ interactions and, consequently, a lower *M_S_* value [9].

The remanent magnetization (*M_R_*) for the undoped CoFe_2_O_4_ calcined at 700 °C is 3.5 emu/g. The doping with monovalent cations (Ag^+^, Na^+^) increases the *M_R_* to 7.7–7.9 emu/g, while doping with the divalent (Ca^2+^, Cd^2+^) and trivalent (La^3+^) cations decreases the *M_R_* to 1.8–3.0 emu/g. In the samples calcined at 1000 °C, the *M_R_* of the doped ferrites increases, compared to the *M_R_* of CoFe_2_O_4_ (13.4 emu/g), except for the *M_R_* of Na_0.1_Co_0.95_Fe_2_O_4_, which slightly decreases (11.3 emu/g).

The slight decrease in the *Hc* values in the doped samples could result from the magnetocrystalline anisotropy, microstrain, size distribution and the decrease in the magnetic domain size [27]. For the gels calcined at 700 °C, the *H_C_* of the undoped CoFe_2_O_4_ is 600 Oe, while that of the doped ferrites is lower, which is most probably due to changes in the crystallite size, anisotropy and formation of agglomerates that increase the average particle size above the critical single-domain, which results in a multi-domain structure and the reduction in pinning effects on the domain wall mobility at the grain boundary [22,55]. Oppositely, for the gels calcined at 1000 °C, the *H_c_* of the doped CoFe_2_O_4_ with monovalent cations (Ag^+^ si Na^+^) are comparable, while for those doped with the di- and trivalent cations, the *H_c_* is lower than that of the undoped CoFe_2_O_4_ (1750 Oe). The lower *H_c_* values of the obtained gels indicate a spin distortion on the surface, owing to the magnetocrystalline anisotropy [24]. The presence of SiO_2_ generates stress on the surface of the ferrite particles, which hinders the rotation of the dead layer’s magnetic moments and contributes to the reduction in the *H_c_* [21,22]. The increase in the surface potential barrier caused by crystalline lattice defects, such as the deviation of atoms from the normal positions in the surface layers, also determines the increase in the *H_C_* [29]. 

The *n_B_* of the gels calcined at 700 °C decreases from 0.935 (CoFe_2_O_4_) to 0.857–0.815 by doping. Additionally, in the samples calcined at 1000 °C, except for doping with Cd^2+^ and La^3+^, the *n_B_* of the doped samples is lower than that of the undoped CoFe_2_O_4_ (0.977).

To calculate the magnetic anisotropy constant (*K*), we assumed that the spinel ferrite particles have a spherical shape. The value of *K* depends on the crystalline symmetry of the lattice, the crystalline anisotropy, and the particle size and shape [21]. The highest *K* was obtained for the undoped CoFe_2_O_4_ (1.13·10^−3^ erg/cm^3^ in gels calcined at 700 °C, and 3.46·10^−3^ erg/cm^3^ in gels calcined at 1000 °C). The value of *K* increases with the increasing calcination temperature and decreases by doping. A possible explanation for this decrease could be the pinning of some surface spins in the magnetically disordered surface layer, which needs a higher magnetic field for magnetic saturation [22]. In addition, the magnetic disorder may originate in randomly oriented grains of different sizes and disordered vacancies [22]. The individual *K* of particles acts as an energy barrier and delays the switch of the magnetization direction to the easy axis [22]. Crystalline anisotropy is strongly affected by the volume strain in the crystal, which is determined by the substitution of Fe^3+^ ions by the different sized (La^3+^) ions [55].

To summarize, the embedding of the undoped and doped CoFe_2_O_4_ in the non-magnetic SiO_2_ matrix promotes both the formation of single-phase spinel and minimization of the spin disorder and surface roughness, thus enhancing the magnetic properties of the ferrites. Combining the best magnetic properties and morphological configuration of the undoped and doped CoFe_2_O_4_ can be of interest for several applications, such as high-density storage and biomedicine. Moreover, since SiO_2_ is non-toxic, biologically inert, and widely accepted material by the living body, and even reduces the inflammatory risk, embedding the undoped and doped CoFe_2_O_4_ could enhance their biocompatibility [21]. Although the properties of the obtained doped CoFe_2_O_4_ could be further improved by optimizing the amount of dopant ions, the calcination temperature, or the SiO_2_-to-ferrite ratio, our study brings valuable baseline data on the properties of doped CoFe_2_O_4_-SiO_2_ nanocomposites.

## 3. Materials and Methods

### 3.1. Reagents

All chemicals and reagents were used as received, without additional purification. Tetrahydrate calcium nitrate (Ca(NO_3_)_2_∙4H_2_O, 99%), silver nitrate (AgNO_3_, 99%), hexahydrate lanthanum nitrate (La(NO_3_)_3_∙6H_2_O, 98%) and tetrahydrate cadmium nitrate (Cd(NO_3_)_2_∙4H_2_O, 99%) were purchased from Carlo Erba (Milan, Italy), while nonahydrate ferric nitrate (Fe(NO_3_)_3_∙9H_2_O, 98%), sodium nitrate (NaNO_3_, 99%), hexahydrate cobalt nitrate (Co(NO_3_)_2_∙6H_2_O, 98%), 1,4-BD 99%, TEOS (99%) and ethanol 96% were purchased from Merck (Darmstadt, Germany).

### 3.2. Synthesis

CoFe_2_O_4_, Ag_0.1_Co_0.95_Fe_2_O_4_, Na_0.1_Co_0.95_Fe_2_O_4_, Ca_0.1_Co_0.9_Fe_2_O_4_, Cd_0.1_Co_0.9_Fe_2_O_4_ and La_0.1_CoFe_1.9_O_4_ embedded in SiO_2_ gels containing 50 wt.% ferrite and 50 wt.% SiO_2_ were prepared through a sol-gel route using different X/Co/Fe (X= Ag, Na, Ca, Cd, La) molar ratios, i.e., 0/1/2 (CoFe_2_O_4_), 1/9.5/20 (Ag_0.1_Co_0.95_Fe_2_O_4_), 1/9.5/20 (Na_0.1_Co_0.95_Fe_2_O_4_), 1/9/20 (Ca_0.1_Co_0.9_Fe_2_O_4_), 1/9/20 (Cd_0.1_Co_0.9_Fe_2_O_4_) and 1/10/19 (La_0.1_CoFe_1.9_O_4_). The sols were prepared by stirring the metal nitrates with 1,4-BD, TEOS and ethanol using a NO_3_^−^/1,4-BD/TEOS molar ratio of 1/1/1. The resulting sols were stirred continuously for 30 min and kept at room temperature until complete gelation; the formed gel enclosed a homogenous mixture of metal nitrates and 1,4-BD. As high-purity gels with large crystallites are obtained by using a thermal pretreatment [5], the obtained gels were ground, heated at 40 °C (2 h) and 200 °C (5 h) and calcined at 400 °C (5 h), 700 °C (5 h) and 1000 °C (5 h) using a Nabertherm LT9 (Lilienthal, Germany) muffle furnace.

### 3.3. Characterization

The reaction progress was investigated via thermogravimetry (TG) and differential thermal analysis (DTA) in air, up to 1000 °C, at 10 °C·min^−1^ using alumina standards and a simultaneous SDT Q600 (TA Instruments, New Castle, DE, USA) thermal analyzer. The Fourier transform infrared (FT-IR) spectra of samples were recorded on KBr pellets containing 1% sample using a BX II FT-IR (Perkin Elmer, Waltham, MA, USA) spectrometer. The crystalline phases were investigated via X-ray diffraction using a D8 Advance (Bruker, Karlsruhe, Germany) diffractometer at ambient temperature with CuKα radiation (λ = 1.5418 Å), operating at 40 kV and 35 mA. The composition of gels calcined at 400, 7000 and 1000 was confirmed via Optima 5300 DV (Perkin Elmer, Norwalk, CT, USA) ICP-OES after microwave digestion using a Speedwave Xpert (Berghof, Germany) system. Atomic force microscopy (AFM) was performed using a JSPM 4210 (JEOL, Tokyo, Japan) microscope in tapping mode using an NSC 15 (Mikromasch, Sofia, Bulgaria) silicon cantilever with a nominal resonant frequency of 325 kHz and a nominal force constant of 40 N/m. Three different 1 µm x 1 µm areas of the thin films obtained by transferring nanoparticles onto glass slides via adsorption from aqueous suspension were scanned for each sample. Image processing and topography were performed using a WinSPM 2.0 software (JEOL, (Tokyo, Japan). Cantilever characteristics were considered in the particle size determination. The particles’ morphology was visualized using an HD-2700 (Hitachi, Tokyo, Japan) transmission electron microscope (TEM). The magnetic measurements were performed using a 7400 vibrating sample magnetometer (VSM) (Lake Shore, Carson, CA, USA). The hysteresis loops were recorded at room temperature, up to an applied field of 2 T, while the magnetization (*M*) was measured in a high magnetic field of up to 5 T. 

## 4. Conclusions

The influence of doping with monovalent (Ag^+^, Na^+^), divalent (Ca^2+^, Cd^2+^), and trivalent (La^3+^) ions on the structural, morphological, surface, and magnetic properties of CoFe_2_O_4_ was investigated. The kinetic formation of the doped and undoped CoFe_2_O_4_ showed that the activation energy of CoFe_2_O_4_ (1.236 kJ/mol) increased to 1.487–1.747 kJ/mol by Ag^+^, Ca^2+^, Cd^2+^, and La^3+^ doping, and decreased to 1.102 kJ/mol by Na^+^ doping, while the rate constant increased with the calcination temperature and depended on the doping ion. Poorly crystalline ferrites at 400 and 700 °C, and a well-crystallized single-phase ferrite in the undoped CoFe_2_O_4_ at 1000 °C, were observed. By doping, besides the well-crystallized ferrite, crystalline silica phases (cristobalite and quartz) were also formed. Although all the obtained gels have a cubic spinel structure, doping with different ions changed in the structural parameters determined via XRD. The AFM revealed that a low calcination temperature generated mainly spherical particles with a polycrystalline structure containing ferrite crystallites mixed with amorphous material. The increase in the calcination temperature led to a larger crystallite size, forming particles by a single-phase ferrite core covered by traces of secondary phases. The TEM measurements also indicate that thermal treatment is the main cause of the large size of the obtained nanoparticles; these results indicate a coalescence of nanoparticles, increasing the mean size. At 700 and 1000 °C, a single magnetic phase is generally observed, except in the case of doping with monovalent (Ag^+^, Na^+^) ions at 1000 °C, when the formation of two magnetic phases is favored. Moreover, the magnetic parameters of the gels calcined at 1000 °C were higher than those at 700 °C. The doping with monovalent ions decreased the *M_S_* and increased the *H_C_*, while the doping with multivalent ions increased the *M_S_* and decreased the *H_C_*. The *K* value decreased with doping, with the undoped CoFe_2_O_4_ displaying the highest anisotropy constant. The obtained results confirm that doping plays an important role in the tuning of the physical properties of promising CoFe_2_O_4_, which may be of great importance in the exploration of new applications in high-density information storage, drug delivery and tissue imaging.

## Figures and Tables

**Table 1:** Rate constant values (*k*) and activation energies (*E_a_*) of CoFe_2_O_4_, Ag_0.1_Co_0.95_Fe_2_O_4_, Na_0.1_Co_0.95_Fe_2_O_4_, Ca_0.1_Co_0.9_Fe_2_O_4_, Cd_0.1_Co_0.9_Fe_2_O_4_ and La_0.1_CoFe_1.9_O_4_ gels heated at 150, 200, 250 and 300 °C.

Gels	*k* (s^−1^)				*E_a_* (kJ/mol)
	150 °C	200 °C	250 °C	300 °C	
**CoFe_2_O_4_**	0.223	0.285	0.330	0.365	1.217
**Ag_0.1_Co_0.95_Fe_2_O_4_**	0.196	0.271	0.336	0.391	1.709
**Na_0.1_Co_0.95_Fe_2_O_4_**	0.205	0.256	0.293	0.320	1.121
**Ca_0.1_Co_0.9_Fe_2_O_4_**	0.222	0.300	0.359	0.404	1.501
**Cd_0.1_Co_0.9_Fe_2_O_4_**	0.184	0.251	0.303	0.341	1.549
**La_0.1_CoFe_1.9_O_4_**	0.194	0.275	0.340	0.391	1.743

**Table 2:** Structural parameters determined via XRD and M/Co/Fe molar ratio of gels calcined at 400, 700 and 1000 °C.

Parameter	Temp (°C)	CoFe_2_O_4_	Ag_0.1_Co_0.95_Fe_2_O_4_	Na_0.1_Co_0.95_Fe_2_O_4_	Ca_0.1_Co_0.9_Fe_2_O_4_	Cd_0.1_Co_0.9_Fe_2_O_4_	La_0.1_CoFe_1.9_O_4_
**D_XRD_ (nm)**	400	13.2	14.0	13.6	14.6	12.3	15.5
	700	23.4	24.7	23.7	25.2	22.5	26.1
	1000	37.2	65.1	47.6	73.7	35.0	81.8
**DC (%)**	400	58.8	62.7	64.2	63.7	64.8	60.2
	700	71.7	65.2	69.6	67.8	72.9	75.4
	1000	85.7	86.9	87.8	92.5	88.9	90.6
**a (Å)**	400	8.391	8.380	8.377	8.374	8.400	8.408
	700	8.395	8.400	8.390	8.388	8.412	8.411
	1000	8.403	8.410	8.400	8.397	8.422	8.423
**V (Å^3^)**	400	590.8	588.5	587.8	587.2	592.7	594.4
	700	591.6	592.7	590.6	590.2	595.2	595.0
	1000	593.3	594.8	592.7	592.1	597.4	597.6
**d_A_ (Å)**	400	3.633	3.629	3.627	3.626	3.637	3.641
	700	3.635	3.637	3.633	3.632	3.643	3.642
	1000	3.639	3.642	3.637	3.636	3.647	3.647
**d_B_ (Å)**	400	2.967	2.963	2.962	2.961	2.970	2.973
	700	2.968	2.970	2.966	2.966	2.974	2.974
	1000	2.971	2.973	2.970	2.969	2.978	2.978
**d_p_ (g/cm^3^)**	400	4.433	4.805	4.472	4.445	4.755	4.685
	700	4.453	4.833	4.494	4.462	4.771	4.706
	1000	4.471	4.861	4.517	4.491	4.794	4.721
**d_XRD_ (g/cm^3^)**	400	5.276	5.473	5.288	5.265	5.445	5.429
	700	5.269	5.435	5.263	5.239	5.422	5.424
	1000	5.253	5.415	5.244	5.222	5.402	5.400
**P (%)**	400	16.0	12.2	15.4	15.6	12.7	13.7
	700	15.5	11.1	14.6	14.8	12.0	13.2
	1000	14.9	10.2	13.9	14.0	11.3	12.6
**M/Co/Fe molar ratio**	400	0/0.95/1.97	0.08/0.91/1.95	0.08/0.91/1.98	0.08/0.86/1.96	0.08/0.84/1.96	0.07/0.94/1.85
	700	0/0.96/1.97	0.08/0.92/1.97	0.08/0.92/1.96	0.08/0.86/1.97	0.08/0.86/1.96	0.07/0.97/1.86
	1000	0/0.99/2.01	0.09/0.94/1.98	0.11/0.96/1.98	0.11/0.89/2.01	0.09/0.89/2.02	0.09/0.98/1.89

**Table 3:** Morphological parameters of gels calcined at 400, 700 and 1000 °C determined using AFM.

Parameter	Temp. (°C)	CoFe_2_O_4_	Ag_0.1_Co_0.95_Fe_2_O_4_	Na_0.1_Co_0.95_Fe_2_O_4_	Ca_0.1_Co_0.9_Fe_2_O_4_	Cd_0.1_Co_0.9_Fe_2_O_4_	La_0.1_CoFe_1.9_O_4_
**D_AFM_ (nm)**	400	25	27	24	28	20	30
	700	30	31	35	33	28	38
	1000	40	70	52	75	39	90
**D_TEM_ (nm)**	1000	38	67	50	76	37	84
**Height (nm)**	400	12	13	10	11	15	9
	700	19	7	25	8	15	12
	1000	41	42	45	37	63	36
**Rg (nm)**	400	0.81	0.95	0.97	0.77	1.10	1.04
	700	1.95	0.83	2.42	0.75	1.50	1.10
	1000	5.80	6.16	6.21	5.35	7.82	5.58
**Powder surface area (nm^2^)**	400	1017	1013	1016	1015	1018	1012
	700	1028	1021	1032	1023	1024	1026
	1000	1083	1095	1094	1062	1160	1069

**Table 4:** Magnetic parameters of gels calcined at 700 and 1000 °C.

Parameter	Temp (°C)	CoFe_2_O_4_	Ag_0.1_Co_0.95_Fe_2_O_4_	Na_0.1_Co_0.95_Fe_2_O_4_	Ca_0.1_Co_0.9_Fe_2_O_4_	Cd_0.1_Co_0.9_Fe_2_O_4_	La_0.1_CoFe_1.9_O_4_
***M_S_* (emu/g)**	700	30.0	25.4	27.7	26.7	26.2	26.5
	1000	31.5	29.0	31.2	39.4	36.3	36.6
***M_R_* (emu/g)**	700	3.5	7.7	7.9	2.7	3.0	1.8
	1000	13.4	13.8	11.3	14.5	17.0	15.1
***H_c_* (Oe)**	700	600	530	360	410	440	385
	1000	1750	1850	1760	840	1070	1300
***n_B_***	700	0.935	0.815	0.857	0.821	0.831	0.821
	1000	0.977	0.814	0.965	0.917	1.151	1.175
***K*** **·10^3^** **(erg/cm^3^)**	700	1.13	0.84	0.63	0.68	0.72	0.64
	1000	3.46	2.90	3.45	2.08	2.44	2.99

## Data Availability

The data presented in this study are available on request from the corresponding author.
